# Supplementary material for: Characteristics of Intestinal Barrier State and Immunoglobulin-Bound Fraction of Stool Microbiota in Advanced Melanoma Patients Undergoing Anti-PD-1 Therapy
Source: Int J Mol Sci. 2025 Aug 20;26(16):8063. doi: 10.3390/ijms26168063 (PMC12387085; doi:10.3390/ijms26168063)
Supplement: Supplementary file 1 [file ijms-26-08063-s001.zip › Supplementary Materials Figures S1-S3.pdf]

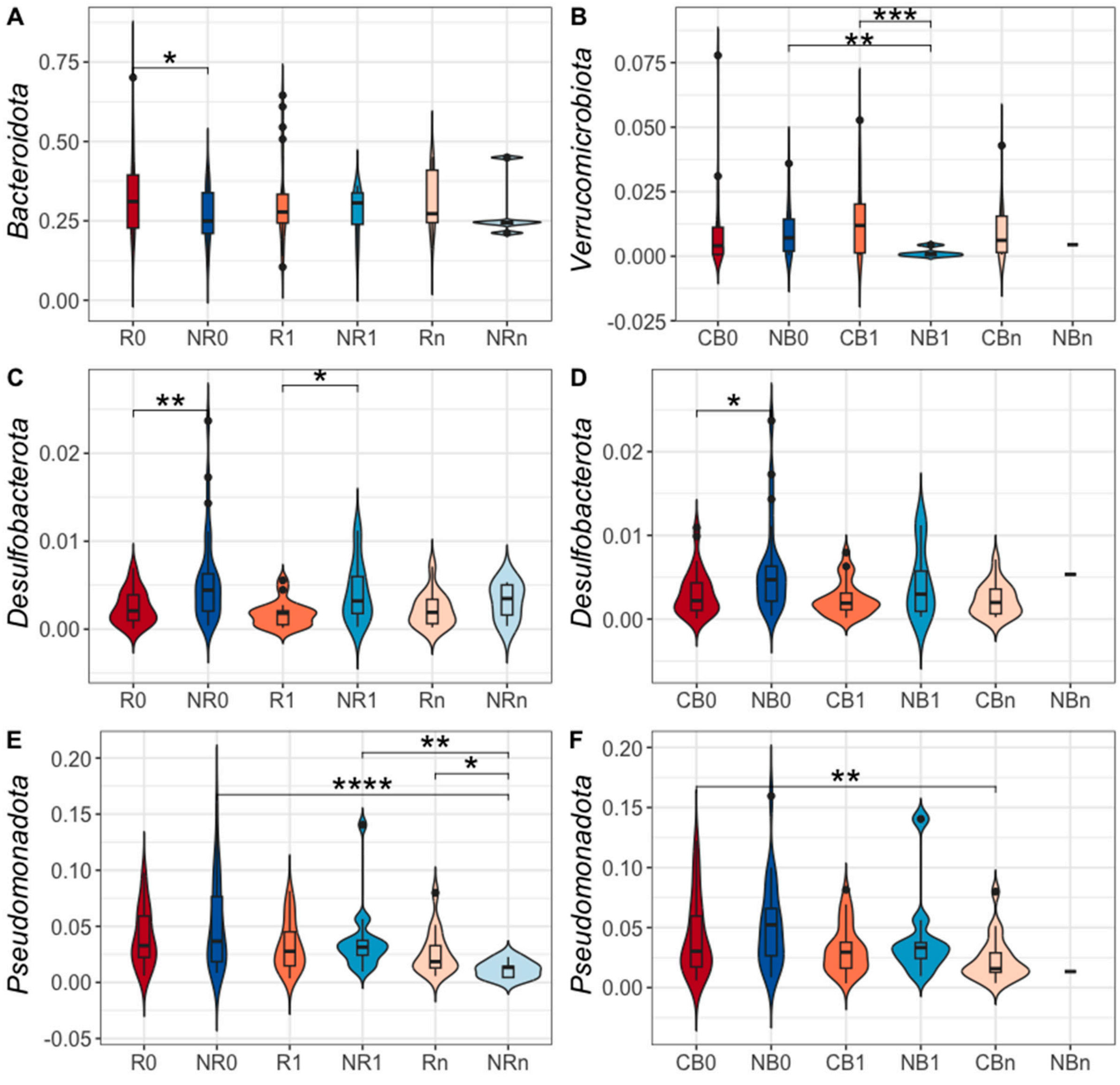

Figure S1. The comparison of the relative abundances of phyla: *Bacteroidota* – formerly *Bacteroidetes* (A), *Verrucomicrobiota* – formerly *Verrucomicrobia* (B), *Desulfobacterota* (C and D), and *Pseudomonadota* – formerly *Proteobacteria* (E and F) in the total stool microbiota between advanced melanoma patients receiving the anti-PD-1 therapy, before the its start at T_0 (0) and during treatment at T_1 and T_n (1 and n, respectively). Patients were classified as responders – R or non-responders – NR (A, C, and E) and patients with clinical benefit – CB or patients with no clinical benefit – NB (B, D, and F) according to the clinical outcome of the immunotherapy. The *p*-values describing the statistical significance of the differences in the relative abundances of particular phyla between study subgroups were calculated with the Student's t-test. The p-value ≤ 0.05 was regarded as significant (*: p-values ≤ 0.05, **: p-values ≤ 0.01, ***: p-values ≤ 0.001, ****: p-values ≤ 0.0001). Statistics indicated significant differences in the relative abundances of particular phyla between subgroups of advanced melanoma patient with distinct clinical outcomes of anti-PD-1 therapy and changes in the relative abundance patterns during treatment. In detail, there was a higher relative abundance of *Bacteroidota* phylum members in the R vs. NR at T_0, *Verrucomicrobiota* phylum members in CB vs. NB at T_1, *Desulfobacterota* phylum members in NR vs. R at T_0 and T_1, and in NB vs. CB at T_0, and *Pseudomonadota* phylum members in R vs. NR at T_n. Moreover, the relative

abundance of *Verrucomicrobiota* phylum members decreased in the NB subgroup at T_1, and *Pseudomonadota* phylum members decreased at T_n in the NR and CB subgroups (NR0 vs. NRn, NR1 vs. NRn, and CB0 vs. CBn, respectively). PD-1 – programmed cell death protein 1.

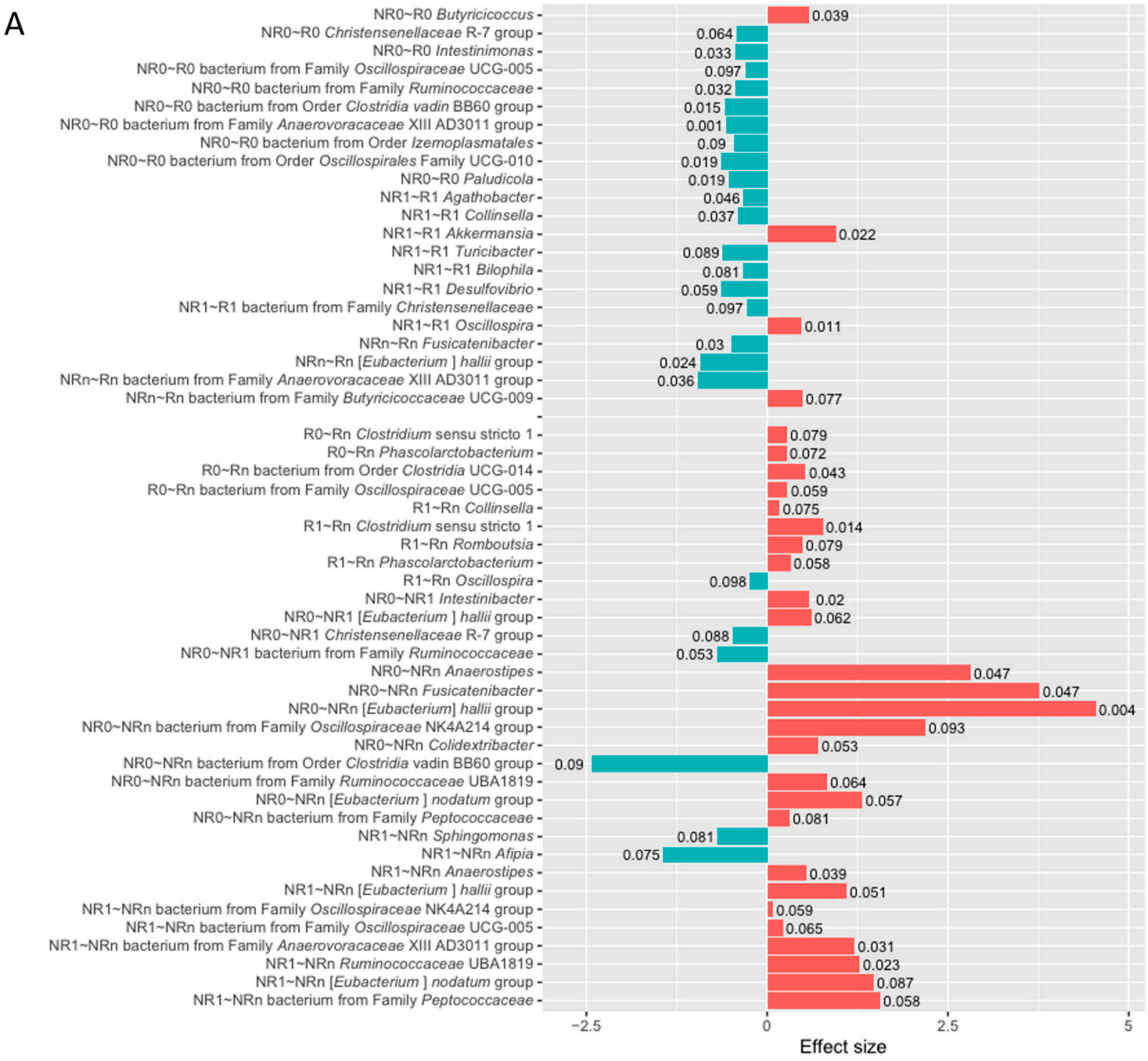

Figure S2. Cont.

B

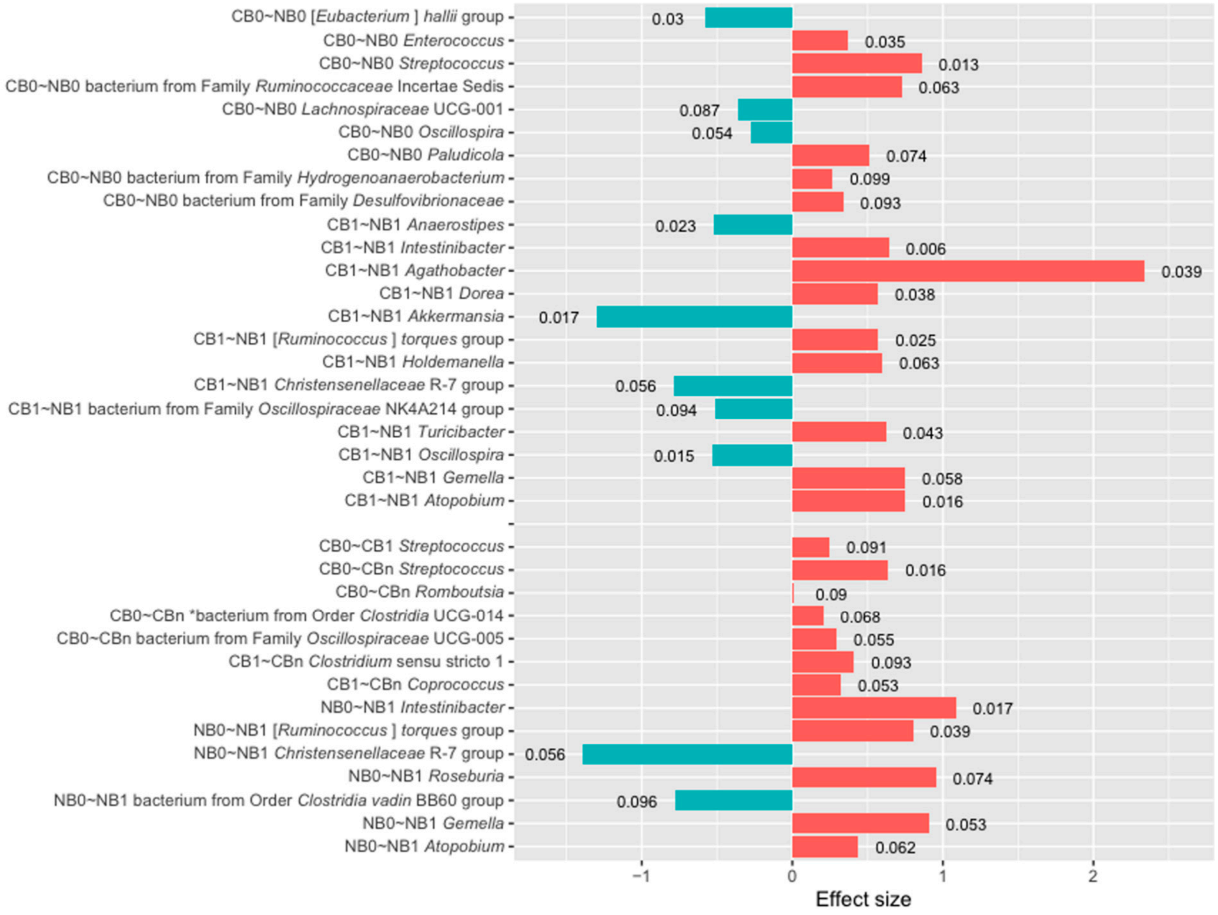

Figure S2. The differentially abundant genera in the total stool microbiota between advanced melanoma patients with favorable vs. unfavorable clinical outcomes of the anti-PD-1 therapy, before its start – at T_0 (0) and during treatment – at T_1 and T_n (1 and n, respectively) identified in the differential abundance analysis (DAA) performed using ANOVA-like Differential Expression version 2 (ALDEx2) tool. Moreover, changes in the relative abundances of genera during treatment (T_0 vs. T_1, T_0 vs. T_n, and T_1 vs. T_n) within those subgroups were also indicated with the DAA. Patients were classified as responders – R or non-responders – NR (A) and as patients with clinical benefit – CB or patients with no clinical benefit – NB (B) according to the clinical outcome of the immunotherapy. The figures illustrate only the statistically significant results (The Wilcoxon Rank Sum Test, *p*-value < 0.1 was regarded as significant). The *p*-values describing the statistical significance of the DAA results were placed at the tips of the bars. The direction of changes in the relative abundances of taxa between the two subgroups being compared was assessed based on the effect size values. The first group of the two being compared is considered a reference group, whereas the second one is a tested group (group designations are placed on the left side of the graph at the beginning of the following lines; 'reference group~tested group'). A positive effect size (red bars) suggests a higher relative abundance of a particular taxon in the tested group compared to the reference group, while negative (blue bars) – lower. Effect size also measures the biological significance of the observed differences (the larger the effect size, the more substantial the difference between subgroups). The names of the differentially abundant taxa are placed on the left side of the graph (next to the subgroup designation). The DAA indicated that the total stool microbiota signatures were associated with clinical outcomes of the anti-PD-1 therapy and have changed during treatment. PD-1 – programmed cell death protein 1.

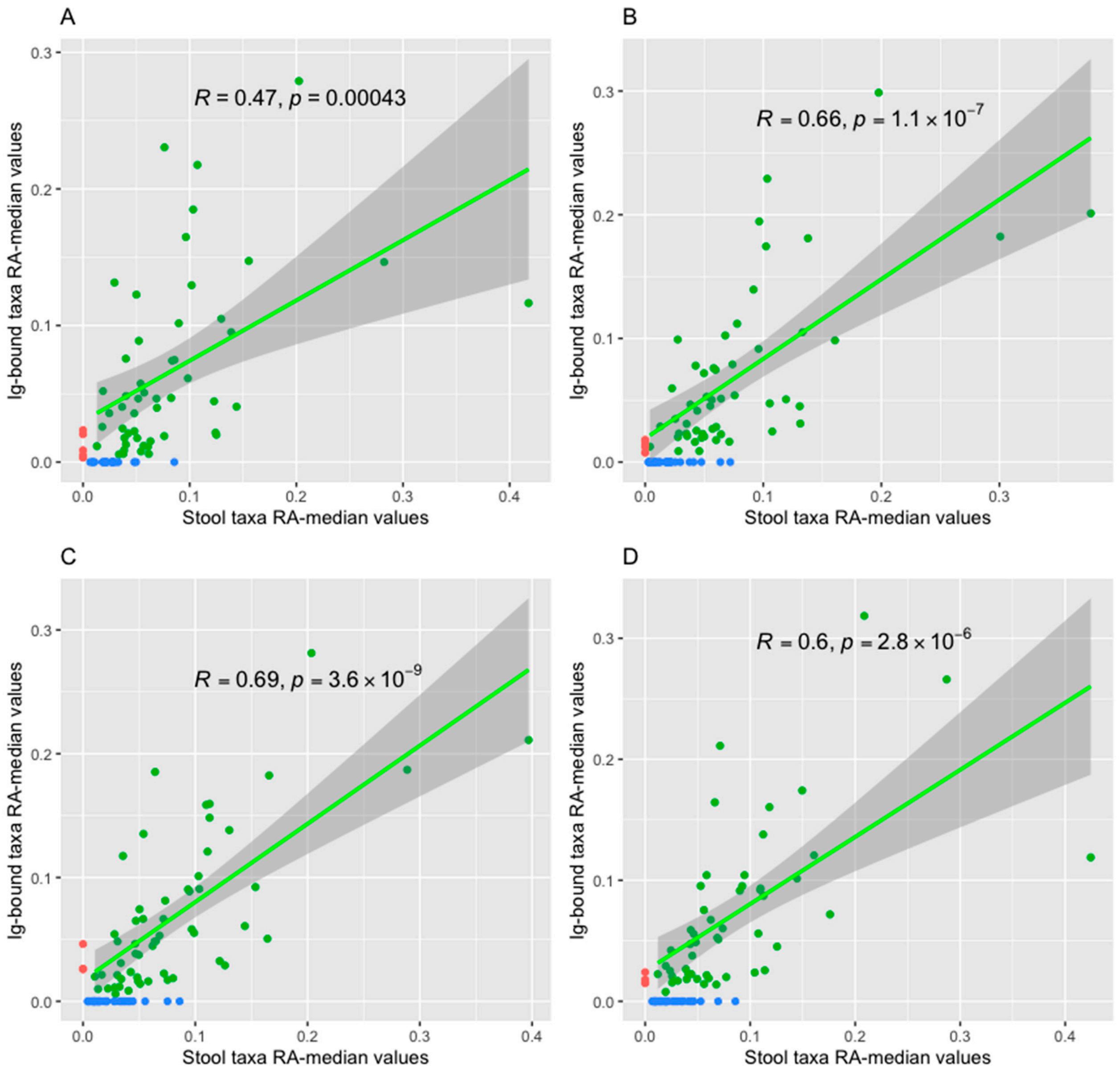

Figure S3. The correlations between the relative abundance (RA) median values of bacteria (at the genus level) detected in the immunoglobulin (Ig)-bound stool microbiota fraction and total stool microbiota (green dots and line) were analyzed in advanced melanoma patients, who responded to the anti-PD-1 therapy – R (A and B) and non-responders – NR (C and D), before its start – at T_0 (0; A and C) and during treatment – at T_1 (1; B and D). Statistics were performed using the Spearman's Rank Correlation Test. The *p*-value ≤ 0.05 was regarded as statistically significant. Figures S3A and B show the trends observed in the R subgroup at T_0 and T_1, respectively; S3C and D – in the NR0 and NR1 subgroups, respectively. Genera detected only in the total stool microbiota (blue dots) or in the Ig-bound stool microbiota fraction (red dots) were also indicated in the figures. The figures demonstrate positive correlations between the RA median values of genera in the Ig-bound stool microbiota and total stool microbiota in the R and NR subgroups at T_0 and T_1. In responders, there was an increase in the Spearman's ρ value during treatment, while in non-responders, there was an opposite trend. PD-1 – programmed cell death protein 1.
